# The mucosal barrier and anti-viral immune responses can eliminate portions of the viral population during transmission and early viral growth

**DOI:** 10.1371/journal.pone.0260010

**Published:** 2021-12-02

**Authors:** Ryan V. Moriarty, Athena E. Golfinos, Dane D. Gellerup, Hannah Schweigert, Jaffna Mathiaparanam, Alexis J. Balgeman, Andrea M. Weiler, Thomas C. Friedrich, Brandon F. Keele, Miles P. Davenport, Vanessa Venturi, Shelby L. O’Connor

**Affiliations:** 1 Department of Pathology and Laboratory Medicine, University of Wisconsin-Madison, Madison, Wisconsin, United States of America; 2 Microbiology Doctoral Training Program, University of Wisconsin-Madison, Madison, Wisconsin, United States of America; 3 Wisconsin National Primate Research Center, Madison, Wisconsin, United States of America; 4 Department of Pathobiological Sciences, University of Wisconsin-Madison, Madison, Wisconsin, United States of America; 5 AIDS and Cancer Virus Program, Frederick National Laboratory, Frederick, MD, United States of America; 6 Infection Analytics Program, Kirby Institute for Infection and Immunity, UNSW Sydney, Sydney, NSW, Australia; University of Pittsburgh, UNITED STATES

## Abstract

Little is known about how specific individual viral lineages replicating systemically during acute Human Immunodeficiency Virus or Simian Immunodeficiency Virus (HIV/SIV) infection persist into chronic infection. In this study, we use molecularly barcoded SIV (SIVmac239M) to track distinct viral lineages for 12 weeks after intravenous (IV) or intrarectal (IR) challenge in macaques. Two Mafa-A1*063+ cynomolgus macaques (*Macaca fascicularis*, CM) were challenged IV, and two Mamu-A1*001+ rhesus macaques (*Macaca mulatta*, RM) were challenged IR with 200,000 Infectious Units (IU) of SIVmac239M. We sequenced the molecular barcode of SIVmac239M from all animals over the 12 weeks of the study to characterize the diversity and persistence of virus lineages. During the first three weeks post-infection, we found ~70–560 times more unique viral lineages circulating in the animals challenged IV compared to those challenged IR, which is consistent with the hypothesis that the challenge route is the primary driver restricting the transmission of individual viral lineages. We also characterized the sequences of T cell epitopes targeted during acute SIV infection, and found that the emergence of escape variants in acutely targeted epitopes can occur on multiple virus templates simultaneously, but that elimination of some of these templates is likely a consequence of additional host factors. These data imply that virus lineages present during acute infection can still be eliminated from the systemic virus population even after initial selection.

## Introduction

While many studies have thoroughly examined the number and diversity of unique viral lineages during acute HIV/SIV infection, few studies focus on the persistence of specific individual viral lineages within a host over time. Defining the number and diversity of these lineages may shed light on the natural progression of how an original viral lineage persists and evolves in the absence of interventions and treatments. In HIV, intravenous (IV) infection is associated with the transmission of numerous unique systemic viral lineages. Researchers have modeled this in SIV studies, where the number of uniquely replicating viruses in plasma correlated with the infectious inoculum dose [1–3].

The molecular tools to characterize viral transmission and evolution have improved over the last ten years. One widely used tool to study the transmission and evolution of HIV and SIV is Single Genome Amplification (SGA) (Reviewed in [4]). However, SGA is expensive and time-intensive, making large-scale studies using SGA quite challenging [5,6]. Instead, viruses containing small molecular tags within their genomes have been developed for animal studies of infectious pathogens. These viruses can be enumerated more efficiently in the blood and tissues with deep sequencing [2,3,7–12]. In contrast to the small number of unique viral templates that can be sequenced by SGA, thousands of barcodes can be deep sequenced from a large population to improve the detection of low-proportion viral variants. These “pseudo-swarm” viruses are isogenic outside the molecular barcode but highly diverse within the barcode, providing each viral lineage with similar fitness *in vitro* and *in vivo* [2,3].

SIVmac239M is a barcoded virus stock derived from clonal SIVmac239 (Nef-open) containing nearly 10,000 viral variants differing only by the 34bp insert [2,3]. Previous studies have used SIVmac239M to understand viral reservoirs in SIV infection [1,2], measure reactivation rates following antiretroviral treatment (ART) interruption [2], enumerate the number of viral lineages during acute infection after IV challenge [2,3], and assess acute immune escape in multiple SIV viral lineages [8]. However, these studies challenged animals IV with SIVmac239M rather than mucosally, even though mucosal transmission is the primary route of HIV transmission. SIVmac239X, a predecessor to SIVmac239M, was previously used to determine the number of transmitted/founder lineages following intrarectal (IR) infection [1] and assess viral dynamics following intravaginal infection [13], but this virus stock contains only 10 distinct lineages, precluding its usefulness in evaluating lineage-specific viral dynamics.

In this study, we compared the number of unique viral lineages circulating systemically during acute infection of macaques challenged either mucosally via IR challenge or systemically via IV challenge with 200,000IU of SIVmac239M. We also examined the relationship between early immune escape and the number of persistent barcoded viral lineages. We followed animals for 12 weeks to determine whether the same viral lineages persisted in the blood throughout this time, as well as the associated variants in acutely targeted T cell epitopes.

## Materials and methods

### Research animals

Two cynomolgus macaques (CMs; *Macaca fascicularis*) and two rhesus macaques (RMs; *Macaca mulatta*) were housed and cared for at the Wisconsin National Primate Research Center (WNPRC) in Madison, WI. All animals were cared for under IACUC protocol #G00680, approved by the University of Wisconsin Graduate School Institutional Animal Care and Use Committee. All procedures, including blood draws and SIVmac239M inoculation, were performed under anesthesia, and we made every effort to minimize distress and suffering. According to previous methods, the RMs were positively genotyped for MHC class I allele Mamu-A1*001 [14], and the CMs were positively genotyped for Mafa-A1*063 [15]. Both RMs (r10001 and r04103) were challenged intrarectally under anesthesia with 200,000 Infection Units (IU) (or 4.89 billion viral copies (vc)) of SIVmac239M, and the CMs (cy0575 and cy0428) were challenged IV with 200,000 IU of SIVmac239M.

All animals were cared for by the staff at the Wisconsin National Primate Research Center (WNPRC) in accordance with the regulations, guidelines, and recommendations outlined in the Animal Welfare Act, the Guide for the Care and Use of Laboratory Animals, and the Weatherall Report. The University of Wisconsin-Madison (UW-Madison), College of Letters and Science and Vice Chancellor for Research and Graduate Education Centers Institutional Animal Care and Use Committee approved the nonhuman primate research covered under protocol G00680. The University of Wisconsin-Madison Institutional Biosafety Committee approved this work under protocol B00000205. All macaques were housed in standard stainless steel primate enclosures providing required floor space and fed using a nutritional plan based on recommendations published by the National Research Council. Macaques had visual and auditory contact with each other in the same room. Housing rooms were maintained at 65–75°F, 30–70% humidity, and on a 12:12 light-dark cycle (ON: 0600, OFF: 1800). Animals were fed twice daily a fixed formula, extruded dry diet with adequate carbohydrate, energy, fat, fiber, mineral, protein, and vitamin content (Harlan Teklad #2050, 20% protein Primate Diet, Madison, WI) supplemented with fruits, vegetables, and other edible objects (e.g., nuts, cereals, seed mixtures, yogurt, peanut butter, popcorn, marshmallows, etc.) to provide variety to the diet and to inspire species-specific behaviors such as foraging. To further promote psychological well-being, animals were provided with food enrichment, structural enrichment, and/or manipulanda. Environmental enrichment objects were selected to minimize chances of pathogen transmission from one animal to another and from animals to care staff. While on study, all animals were evaluated by trained animal care staff at least twice daily for signs of pain, distress, and illness by observing appetite, stool quality, activity level, physical condition. Animals exhibiting abnormal presentation for any of these clinical parameters were provided appropriate care by attending veterinarians. Prior to all minor/brief experimental procedures, macaques were anesthetized with an intramuscular dose of ketamine (10 mg kg^-1^) and monitored regularly until fully recovered from anesthesia. Per WNPRC standard operating procedure (SOP), all animals received environmental enhancement included constant visual, auditory, and olfactory contact with conspecifics, the provision of feeding devices which inspire foraging behavior, the provision and rotation of novel manipulanda (e.g., Kong toys, nylabones, etc.), and enclosure furniture (i.e., perches, shelves). At the end of the study, euthanasia was performed following WNPRC SOP as determined by the attending veterinarian and consistent with the recommendations of the Panel on Euthanasia of the American Veterinary Medical Association. Following sedation with ketamine (at least 15mg/kg body weight, IM), animals were administered at least 50 mg/kg IV or intracardiac sodium pentobarbital, or equivalent, as determined by a veterinarian. Death was defined by stoppage of the heart, as determined by a qualified and experienced individual.

### Viral load quantification

Viral loads (VLs) were measured by the Virology Services unit of the WNPRC longitudinally throughout SIVmac239M infection. VLs were reported from the WNPRC as log viral copies (ceq) per milliliter of plasma. Viral RNA was isolated from plasma samples using the Maxwell 16 Viral Total Nucleic Acid Purification Kit on the Maxwell 16 MDx instrument (Promega, Madison WI). Viral RNA was then quantified using a highly sensitive QRT-PCR assay based on the one developed by Cline et al. [16]. RNA was reverse transcribed and amplified using the Superscript III Platinum One-Step qRT-PCR kit (Invitrogen) on the LightCycler 480 or LC96 instrument (Roche, Indianapolis, IN) and quantified by interpolation onto a standard curve made up of serial tenfold dilutions of *in vitro* transcribed RNA. RNA for this standard curve was transcribed from the p239gag_Lifson plasmid kindly provided by Dr. Jeffrey Lifson, NCI/Leidos. The final reaction mixtures contained 5 mM MgSO4, 150 ng random primers (Promega, Madison, WI), 600 nM each primer, 100 nM probe, and 0.8 μl enzyme mix. Primer and probe sequences are as follows: forward primer: 5’- GTCTGCGTCATCTGGTGCATTC, reverse primer: 5′-CACTAGCTGTCTCTGCACTATGTGTTTTG-3′ and probe: 5′-6-carboxyfluorescein-CTTCCTCAGTGTGTTTCACTTTCTCTTCTGCG-BHQ1-3’. The reactions cycled with the following conditions: 37°C for 15 min, 50°C for 30 min, 95°C for 2 min followed by 50 cycles of 95°C for 15 seconds and 62°C for 1 min. The limit of detection of this assay is 100 copies/ml.

### Isolation of nucleic acids for sequencing

vRNA was isolated from either 500μL or 1ml of plasma using the QIAamp MinElute Virus Spin Kit (Qiagen) according to the manufacturer’s protocol. Viral RNA was eluted from the column in Buffer AVE.

### Deep sequencing of the barcode from plasma viral RNA

5μL of vRNA was reverse-transcribed using SuperScript III One-Step RT-PCR System with forward primer 5’-GCTTTACAGCGGGAGAAGTG-3’ and reverse primer 5’-TGCCAAGTGTTGATTATTTGTC-3’. Viral loads ranged from 2.1 x 10^3^−1.51 x 10^8^ CEQ/mL. This step produced an approximate 2,250bp cDNA amplicon that included the 34-nucleotide barcode. Then, 1-2ng of the purified 2,250 bp amplicon was used as a template to generate a smaller amplicon (416 or 499 base pairs) with PCR primers containing Common Sequence (CS) tags derived from the Access Array Barcode Library for Illumina Sequences (Fluidigm Corporation) (S1 Table and S1 Fig) and Fluidigm barcode tags to uniquely identify each amplicon. This amplicon was generated using Phusion High-Fidelity PCR Master Mix with High-Fidelity (HF) Buffer. PCR conditions were as follows: 98°C for 3 minutes (x1); 98°C for 5 seconds, 60°C for 10 seconds, 72°C for 30 seconds (x29); 72°C for 5 minutes (x1). PCR products were quantified using a Qubit Fluorometer (Invitrogen) and normalized to 1 ng/μL. Each PCR product was purified using the MinElute Gel Extraction Kit (Qiagen) and quantified using the Qubit double-stranded DNA (dsDNA) High Sensitivity Assay Kit (Invitrogen). Samples were pooled and sequenced with a 2 x 300 V3 sequencing kit on an Illumina MiSeq.

### Deep sequencing of the complete SIV genome from plasma

vRNA was isolated from plasma using the MinElute virus spin kit (Qiagen) as described above. 5μL of vRNA was reverse transcribed and amplified using Superscript III one-step RT-PCR system with High-Fidelity Platinum Taq (Invitrogen). This step produced four overlapping amplicons spanning the entire SIV coding sequence, as detailed previously ([17,18] and S1 Fig). PCR products were purified using the MinElute gel extraction kit (Qiagen), then quantified using the Quant-IT double-stranded DNA high-sensitivity (HS) assay kit on a Qubit Fluorometer (Invitrogen). Libraries were generated from 1 ng of pooled amplicons and then tagged using the Nextera XT kit (Illumina). Tagged libraries were quantified using the Quant-IT dsDNA HS assay kit, and average fragment size was determined using an Agilent Bioanalyzer. Libraries were pooled and sequenced on an Illumina MiSeq using either 2 x 250 or 2 x 300 sequencing kits.

### ELISPOT assays

Fresh peripheral blood mononuclear cells (PBMCs) were subjected to an IFN-γ enzyme-linked immunosorbent spot (ELISPOT) assay, as previously described [19]. We briefly blocked a precoated monkey IFN-γ ELISPOTplus plate (Mabtech, Mariemont, OH), and added peptides listed in the figures to each well in duplicate at a final concentration of 1μM. We added 10^5^ PBMCs to each well, and plates were incubated overnight at 37°C. The positive control for this assay was 10μM Concanavalin A, and the plates were developed according to the manufacturer’s protocol (Sigma Aldrich). The wells were imaged with an AID ELISPOT reader (Autoimmun Disgnostika Gmbh). We calculated the number of spot forming cells (SFCs) per 10^6^ PBMCs by subtracting the average number of background spots (average of spots from four wells not stimulated with any peptide, which served as a negative control) and then multiplying this value by 10. We considered a response positive if it exceeded the no stimulation average threshold plus two times the standard deviation of the no stimulation wells or if it had 50 SFCs per 10^6^ PBMCs, whichever was greater. The values for duplicate wells were then averaged and graphed.

### Quality control and analysis of molecular barcode sequences

Paired-end reads from each time point were mapped to SIVmac239M, and we used the read containing the barcode during subsequent steps. We did not use the other read for barcode analysis. The reads containing the barcode were trimmed to Q>15 [20] and analyzed using the Barcode Virus Analysis tool [21], which analyzes barcoded viral lineage populations originating from SIVmac239M data. These methods are further described in Fennessey et al. [2]. For each sample, we calculated the number and frequency of individual viral lineages. We determined the authenticity of viral lineages based on previous studies that extensively tested the SIVmac239M barcode population in the stock and in macaques [2,3]. Samples were excluded if the FASTQ file contained <20,000 reads due to inadequate depth of coverage. All computation and statistical analyses were performed using in-house Python and R code [22].

## Results

### Fewer circulating viral lineages are detectable following mucosal challenge with SIVmac239M compared to intravenous challenge

**Fig 1:**
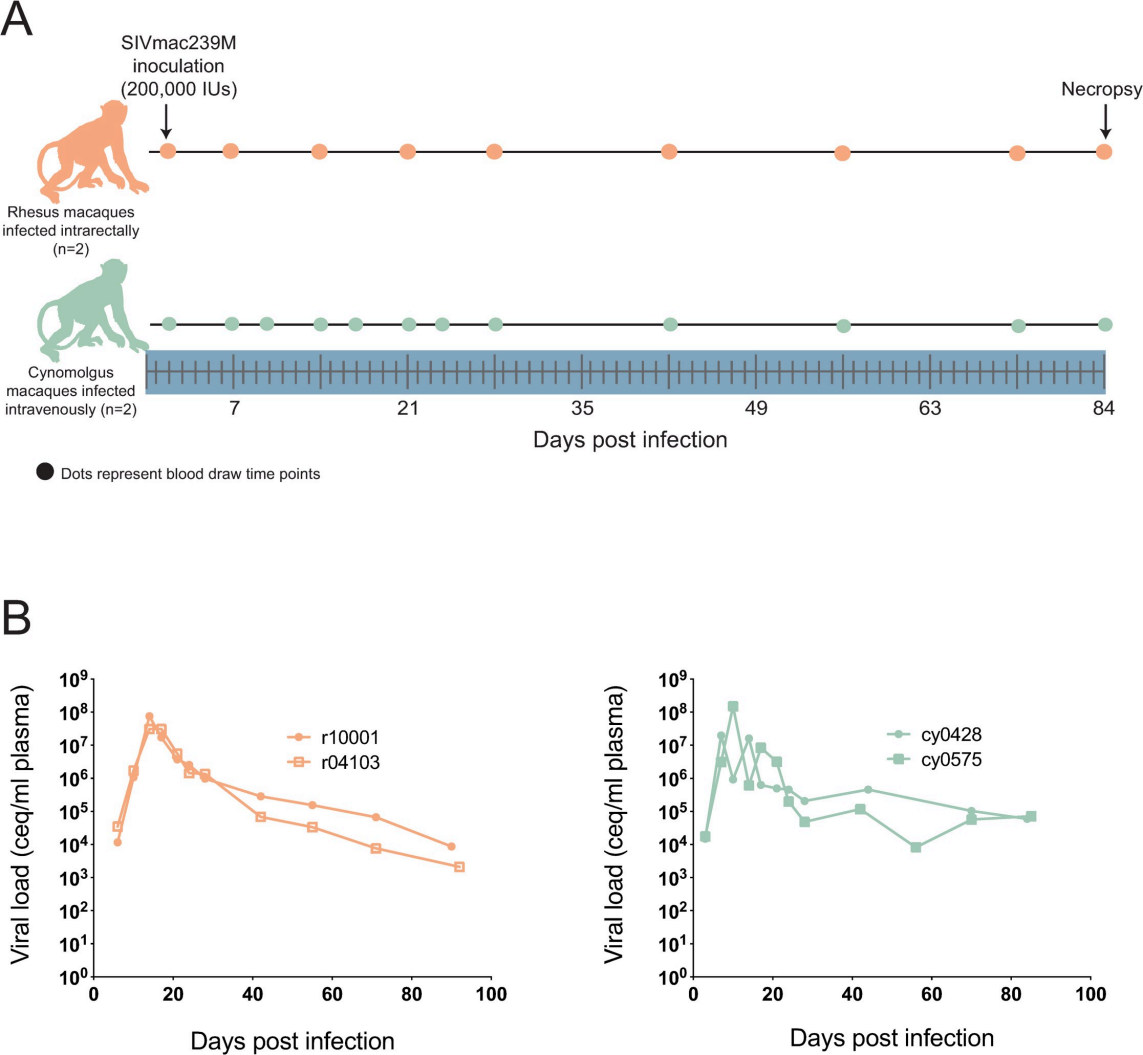
Study timeline and viral loads. A) Two Mamu-A1*001+ rhesus macaques were inoculated intrarectally (IR), and two Mafa-A1*063+ cynomolgus macaques were inoculated intravenously (IV), with 200,000 Infectious Units (IU) of SIVmac239M. Whole blood was drawn at the time points indicated. B) Viral loads in both groups of animals were calculated by qRT-PCR from plasma.

Two Mafa-A1*063+ CMs were challenged IV, and two Mamu-A1*001+ RMs were challenged IR with 200,000 IUs of SIVmac239M. Plasma and PBMC were saved from each animal according to the study timeline (Fig 1A). Peak plasma viral loads (VL) were similar between groups (Fig 1B) and are comparable to peak VLs observed in CMs and RMs with similar MHC genetics in previous studies [23–27]. This observation suggests that regardless of inoculation route, SIVmac239M exhibits similar peak viremia in both groups.

**Fig 2:**
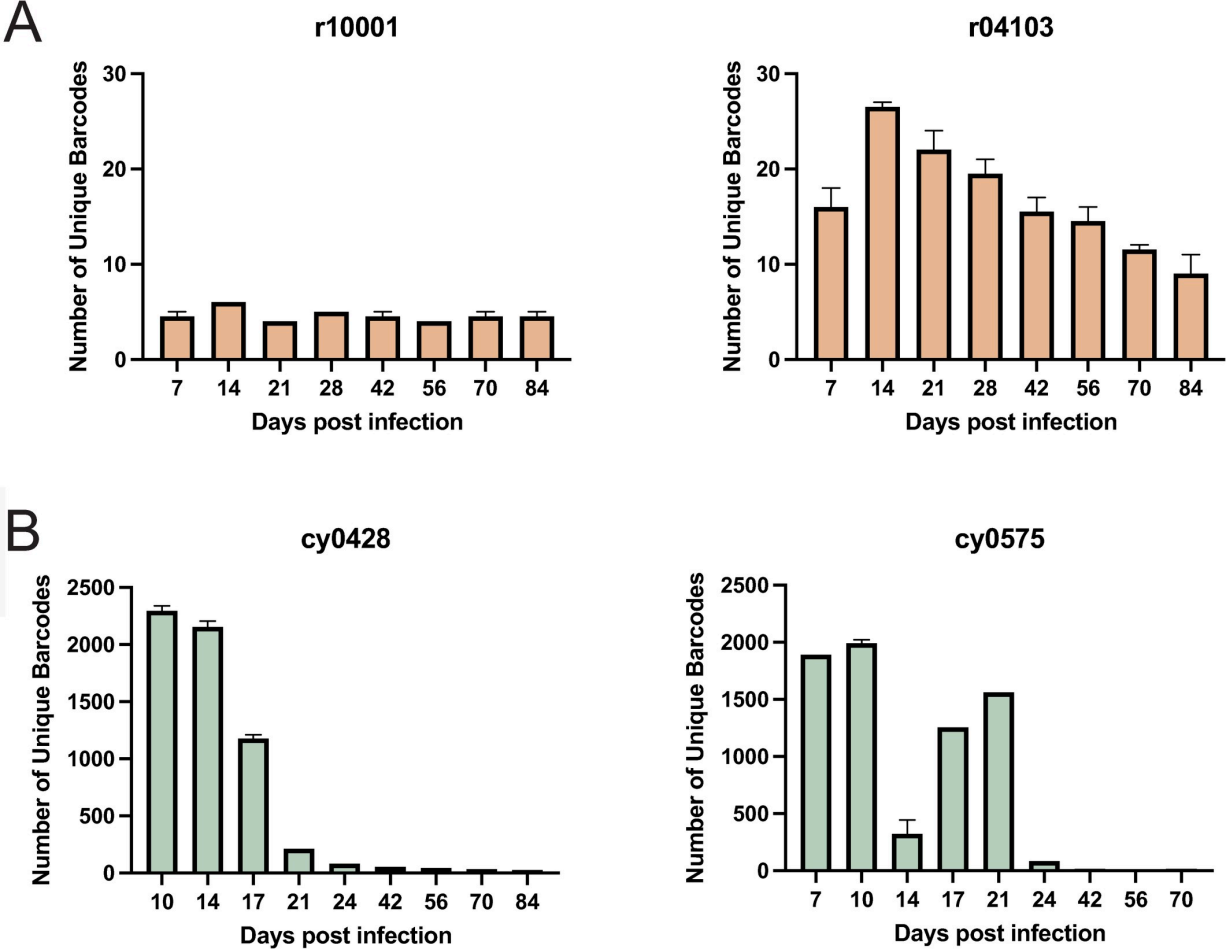
The number of unique viral lineages in IR and IV-challenged macaques. The number of unique viral lineages in intrarectally (IR)-challenged rhesus macaques (A) and intravenously (IV)-challenged cynomolgus macaques (B) between 7 and 84 days post-SIVmac239M infection. When duplicates were present, the median with 95% confidence interval was plotted. When only a single data point was present, that value was plotted.

We assessed the number of unique viral lineages following infection in both groups. We detected between 4 and 6 unique barcoded lineages in r10001, and between 7 and 27 unique viral lineages in r04103 from days 7 to 84 post-infection (Fig 2A). All barcodes were detected by matching to a list of pre-determined barcodes using an R script courtesy of Brandon Keele [21]. If even a single barcode matched the pre-determined barcode list, it was considered present. While the number of unique viral lineages remained relatively consistent in r10001 throughout the infection, r04103 had a higher number of unique circulating viral lineages (range 7–27) that slowly decreased as infection progressed.

In contrast, the number of unique viral lineages detected in the IV-challenged CMs was between 70 and 560-fold higher during the first three weeks of infection than in the IR-challenged RMs. Until day 17, the median number of unique viral lineages across all three time points was 2155 and 1890 for cy0428 and cy0575, respectively. These results were consistent with the number of barcodes previously detected in rhesus macaques challenged intravenously with the same dose of SIVmac239M [3]. The median number of unique viral lineages dropped to 23 and 16 for cy0428 and cy0575, respectively (Fig 2B) from day 24 to the date of necropsy. These data confirm the mucosal bottleneck theory of SIV/HIV transmission since animals infected intrarectally with 200,000 IUs of SIVmac239M do not achieve the same number of circulating viral lineages during acute infection as animals challenged IV with the same dose of SIVmac239M. The average number of barcodes following IV infection ranged between 1890–2239, while the number of barcodes following IR infection ranged between 4 and 27. This suggests that there are between 70-560-fold more detectable barcodes in IV infected animals than in the IR infected animals during the first three weeks of infection.

### Simpson’s Diversity Index of the molecular barcode declines rapidly at three weeks after infection in animals challenged intravenously but not intrarectally

**Fig 3:**
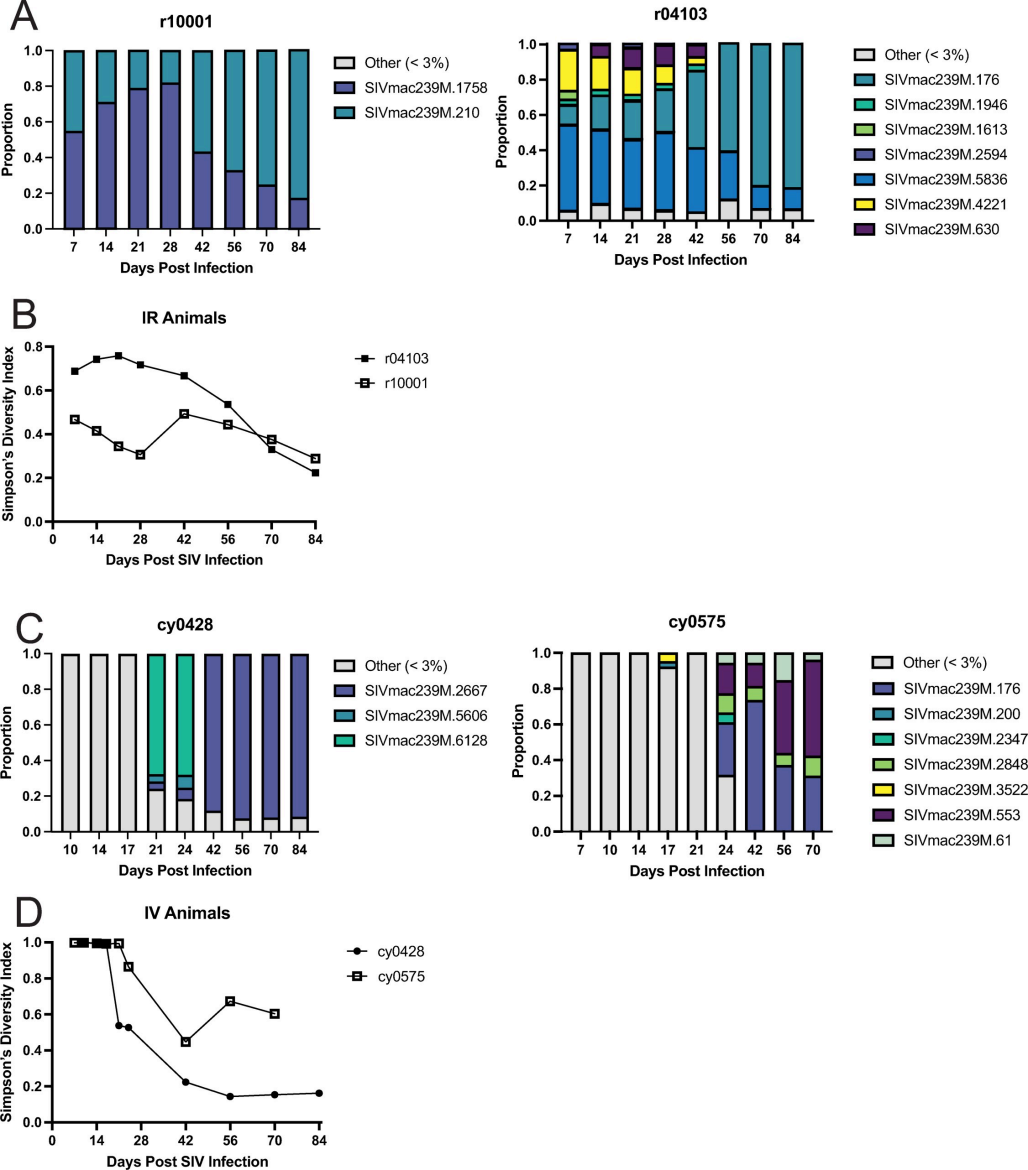
Diversity of the molecular barcodes present in the circulating virus populations. The proportion of individual barcoded lineages circulating in the IR-challenged rhesus (A) and IV-challenged cynomolgus macaques (C) are shown over time. Variants that do not exceed 3% proportion at any point throughout infection were binned into the <3% category. Longitudinal Simpson’s Diversity Index (SDI) of the population of barcoded lineages in the rhesus (B) and cynomolgus macaques (D) was calculated and plotted over time.

We next plotted the frequency of individual barcoded viral lineages comprising the viral population in both IR and IV-challenged animals over time. In both IR-challenged RMs, the relative proportions of each barcode changed slowly over time (Fig 3A), such that one lineage was dominant (>75%) at euthanasia. To quantitatively examine the evenness and diversity of the viral population over time, we used the number and frequency of unique barcoded viral lineages to calculate Simpson’s Diversity Index (SDI) [28]. A population with a high SDI (~1) will have an even distribution of many individual virus lineages in the population. In contrast, a population with a low SDI (~0) likely has a small number of high-frequency viral lineages dominating the population. In the two IR-challenged RMs, the small number of virus lineages comprising the total population was reflected by moderate diversity (SDI 0.5–0.7) during acute infection that slowly declined throughout infection, until an SDI of approximately 0.25 was reached at necropsy (Fig 3B).

Similar to the IR-challenged animals, we characterized the persistence of the individual virus lineages in the IV-challenged animals from three weeks post-infection to necropsy. In stark contrast to the IR-challenged animals, we found a large proportion of low-frequency lineages persisted during the first three weeks of infection, until a single prominent lineage emerged. Barcode 6128 (Fig 3C, left, teal) was present at 68% frequency in cy0428 at days 21 and 24 post-infection, but then disappeared by day 42 and was replaced with barcode 2667 (Fig 3C, left, blue), which remained prevalent until necropsy. Animal cy0575 exhibited more detectable lineages, with five barcodes present at >3% at 24 days post-infection, four of which persisted until necropsy (Fig 3C, right). This difference in population composition is mirrored in the SDI values of the virus populations replicating in both CMs, with the population present in cy0575 consistently having a slightly higher SDI than cy0428 (Fig 3D). We found that in both IV-challenged animals, the first three weeks of infection had a high amount of diversity, represented by a SDI of approximately 1. Consistent with the emergence of a single dominant lineage, diversity began declining starting at day 21, reaching values between 0.4–0.7 and 0.1–0.25 in cy0575 and cy0428, respectively, until necropsy. This decrease is consistent with other studies reporting viral escape from T cells during HIV or SIV infection [8,29–31].

### T cell responses in MHC-restricted epitopes are observed around weeks 3–4 post-infection in all animals

**Fig 4:**
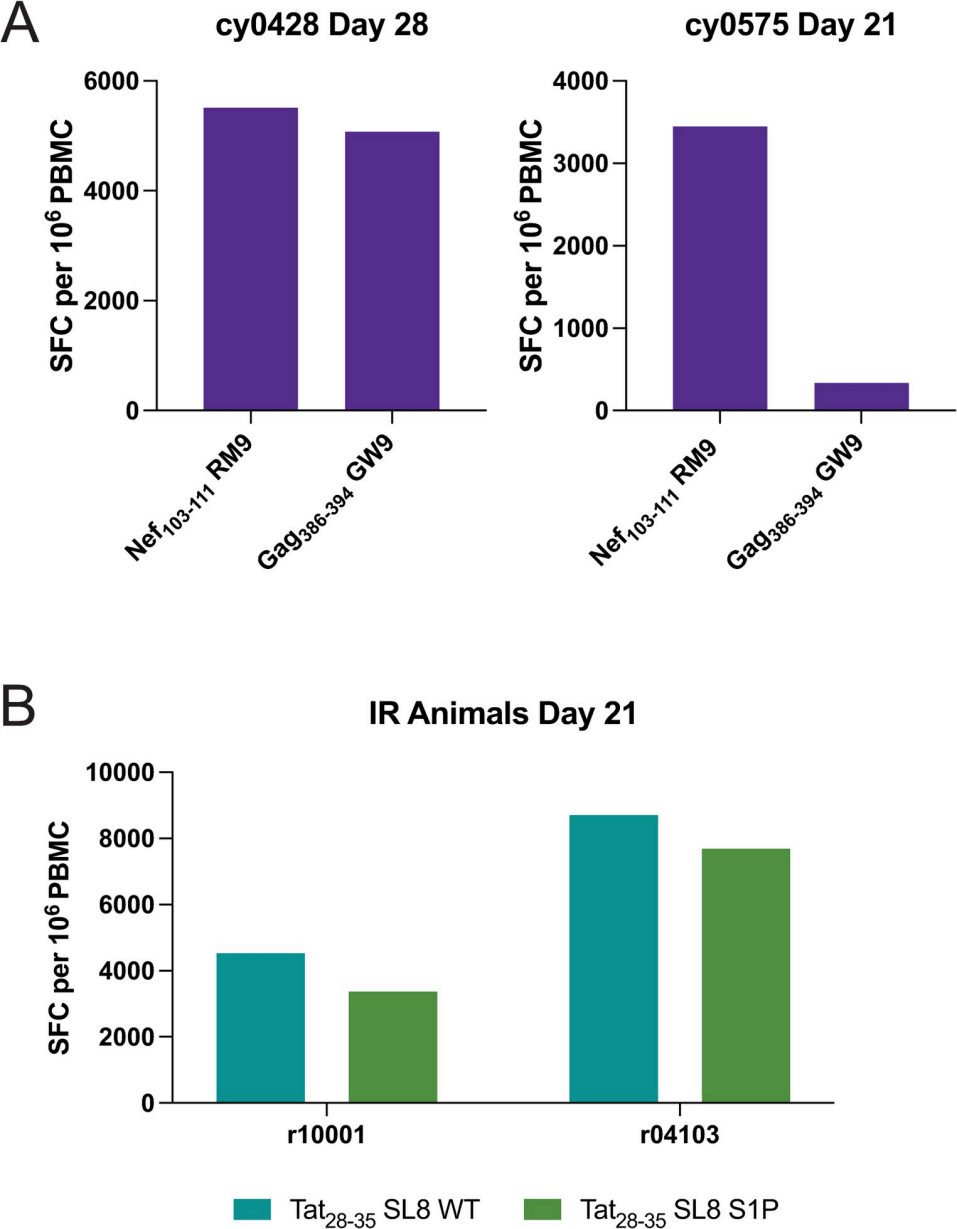
IFNγ-ELISPOT assays detect antigen-specific CD8+ T cells during acute SIV infection. A) In IV-infected cynomolgus macaques, CD8+ T cell responses specific for Nef_103-111_RM9 and Gag_386-394_GW9 at day 28 in cy0428 and day 21 in cy0575 were measured by IFNγ-ELISPOT. B) CD8+ T-cell responses to Tat_28-35_SL8 wild-type (WT) and the S1P mutations assessed at day 21 were measured by IFNγ-ELISPOT in IR-infected rhesus macaques.

SIV-infected Mafa-A1*063+ CMs rapidly develop CD8+ T cells targeting two peptides: Nef_103-111_RM9 and Gag_386-394_GW9 [32,33]. We performed IFNγ-ELISPOT assays using fresh PBMC from cy0428 and cy0575 using these two peptides as stimuli at days 21 or 28 post-infection. We identified detectable responses in the PBMC to the wild type (WT) sequence of both epitopes in both CMs between three- and four-weeks post-infection (Fig 4A), similar to previous studies of CMs [32,33].

Additionally, CD8+ T cells during acute SIV infection of Mamu-A1*001+ RMs target the SIV peptide Tat_28-35_SL8 [8,25,34,35]. We performed IFNγ-ELISPOT assays using fresh PBMC from r10001 and r04103. We detected responses in the PBMC to the WT and S1P variant of Tat_28-35_SL8 on day 21 after infection (Fig 4B), indicating that there were T cell responses specific for both the WT and variant epitope sequences.

### Variation in T cell epitopes is coincident with reduced barcode diversity in IV-challenged CMs

**Fig 5:**
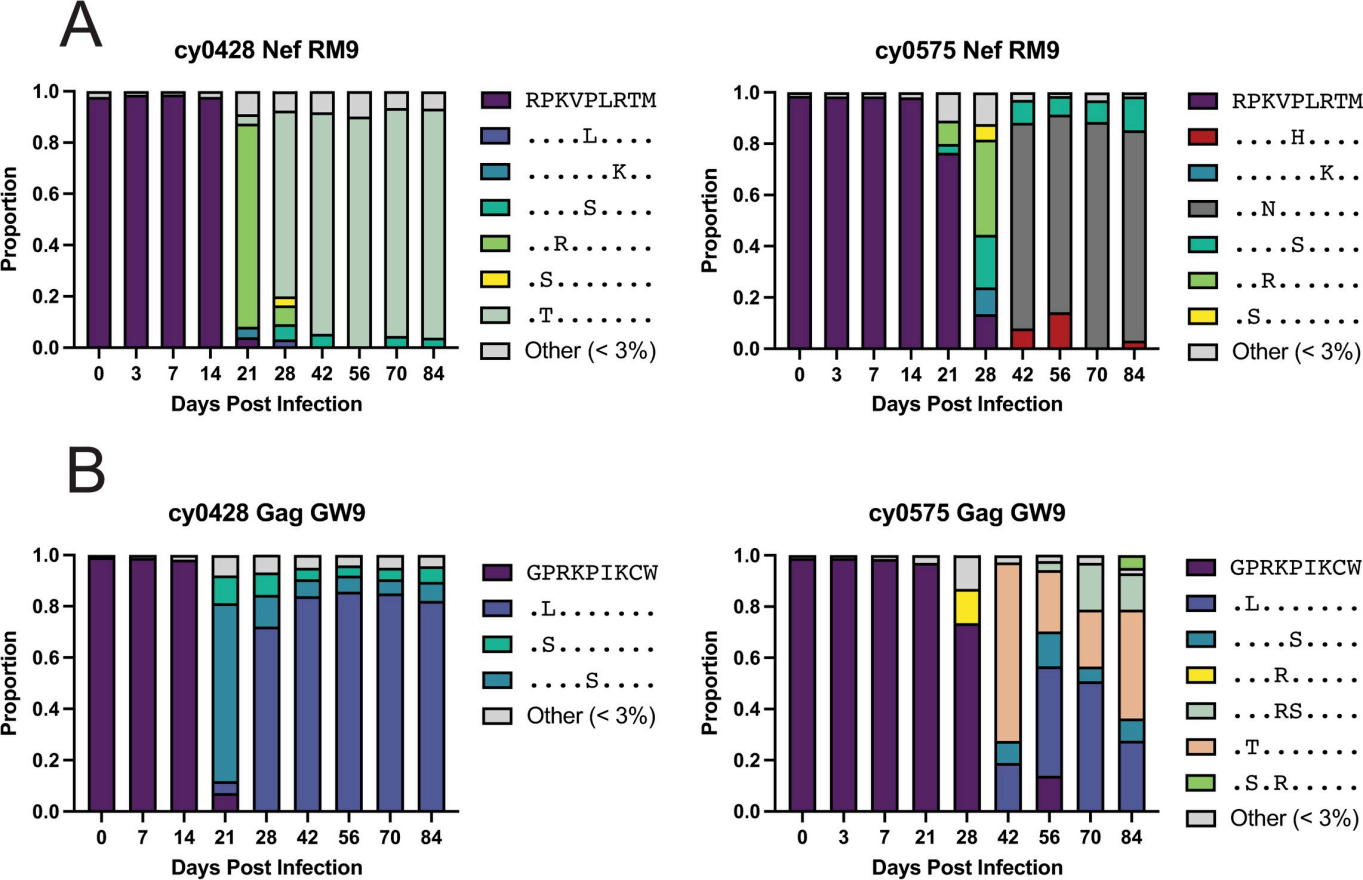
Detection of amino acid variants in Nef_103-111_RM9 and Gag_386-394_GW9 were coincident with reduced barcode diversity. A) Nef_103-111_RM9 variants identified in the intravenously challenged animals cy0428 and cy0575. The wild-type variant is indicated by RPKVPLRTM, shown in dark purple. B) Gag_386-394_GW9 variants identified in the intravenously challenged animals cy0428 and cy0575. The wild-type variant is indicated by GPRKPIKCW, shown in dark purple. Variants comprising less than 3% of the population are binned into the “Other < 3%” category, shown in light grey. Amino acids identical to the wild type are represented with dots, while mutant amino acids are indicated.

We next deep sequenced the Nef_103-111_RM9 and Gag_386-394_GW9 T cell epitopes in viruses circulating in the CMs to determine the frequency of wild type and variant epitope sequences over time. In cy0428, the most dominant Nef_103-111_RM9 variant was a K3R mutant present at 21 days post-infection (Fig 5A, left, green), but this was almost completely replaced with a P2T mutant by day 28 (Fig 5A, left, mint). This variant then dominated the population until necropsy at day 84. In cy0575, we detected five Nef_103-111_RM9 variants >3% beginning at day 28 (Fig 5A, right). However, by day 42 post-infection, the K3N Nef_103-111_RM9 variant (dark grey) was present at greater than 75% frequency and dominated until the animal was euthanized (Fig 5A, right). Interestingly, the K3N variant was comprised of two different nucleotide sequences coding for the same amino acid sequence.

When we assessed Gag_386-394_GW9 variants, we found that both the P2L (blue) and P5S (turquoise) variants were present in both animals after 28 days (Fig 5B). We also found that dominant Gag_386-394_GW9 variants emerged at weeks 21 and 42 days post-infection in CMs cy0428 and cy0575, respectively, similar to the detection of variants in Nef_103-111_RM9 (Fig 5B). Our findings are consistent with our previous studies suggesting that immune escape is conditional [18,33], because the detection of variants in Gag_386-394_GW9 does not occur without variants detectable in Nef_103-111_RM9. Conditional escape requires the presence of an initial mutation for a second mutation to lead to escape within a targeted epitope.

### The same mutations in Tat_28-35_SL8 occur on multiple viral lineages in IR-challenged RMs

**Fig 6:**
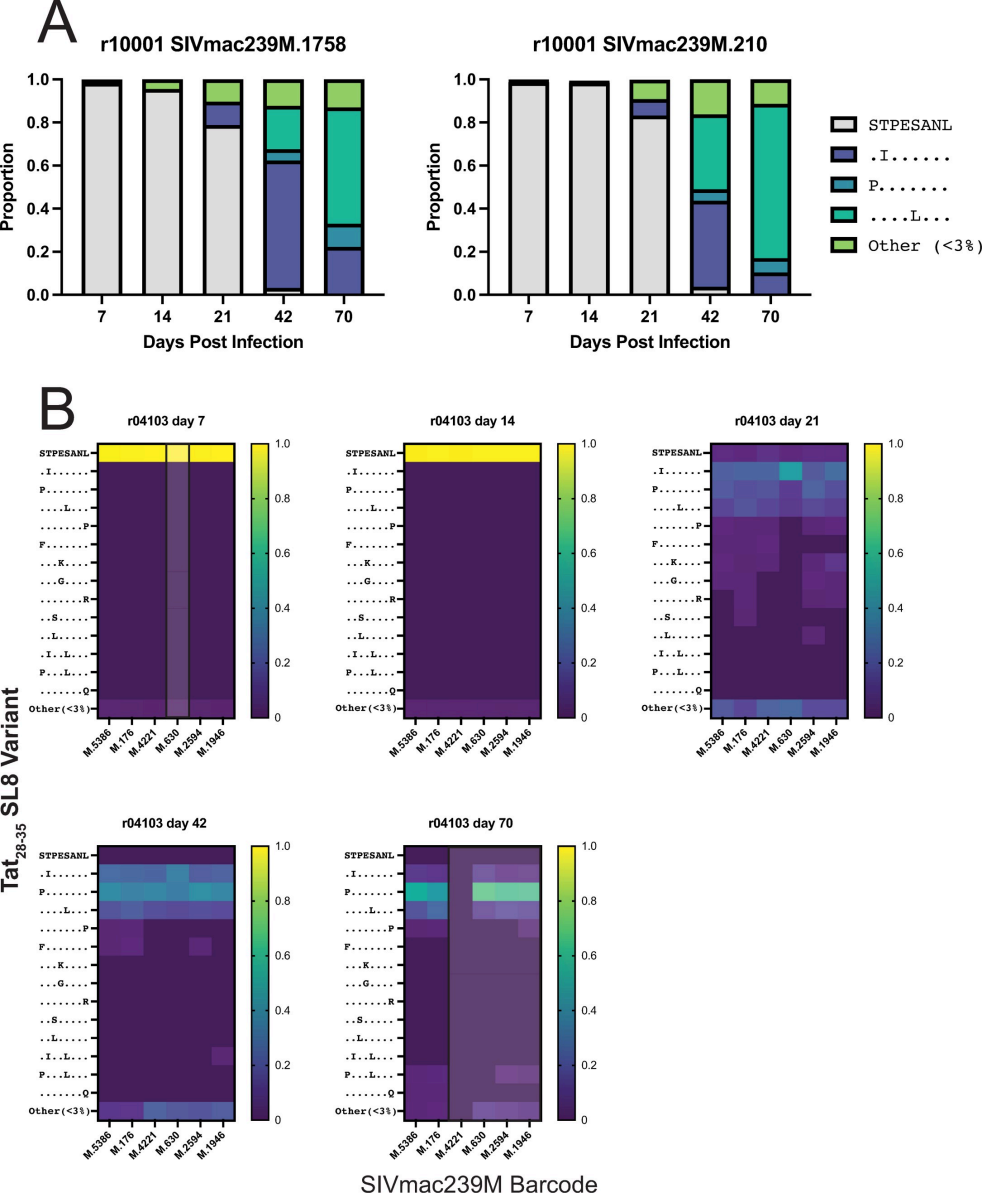
Tat_28-35_SL8 escape variants are linked to multiple viral lineages in intrarectally challenged animals. Amplicon data linking the barcode region to the Mamu-A1*001+ restricted epitope Tat_28-35_SL8 was examined. The frequency of individual epitope variants linked to each barcode lineage is shown for r10001 (A) and r04103 (B). The frequency of each variant in the heat maps (B) is noted in the legend on the right. The wild-type variant is indicated by STPESANL. Amino acids identical to the wild type are represented with dots, while mutant amino acids are indicated. Viral lineages that were present at a frequency of less than 3% in the overall population at a given time point are shaded and outlined. Only viral lineages present at a frequency of 3% or greater than 3% at any time point are shown.

We longitudinally assessed variants in the T cell epitope, Tat_28-35_SL8, targeted in Mamu-A1*001+ RMs. The molecular barcode and Tat_28-35_SL8 were captured on the same amplicon, allowing us to link the sequence of the Tat_28-35_SL8 epitope with the individual virus lineages. In animal r10001, we identified two common viral lineages present throughout infection: 1758 and 210. Both lineages had a similar distribution of Tat_28-35_SL8 variants (Fig 6A) throughout infection, even though lineage 210 became dominant in the plasma (>75%) at necropsy. Although the same Tat_28-35_SL8 variant emerged in both lineages, the expansion of lineage 210 during chronic infection suggests that there are likely other adaptations outside of the Tat_28-35_SL8 sequence contributing to the expansion of this lineage during chronic infection.

The composition of Tat_28-35_SL8 variants replicating in animal r04103 was more complex than for r10001 (Fig 6B). Even though there were multiple viral lineages present during acute infection, lineage 176 was most common at necropsy. We found that the Tat_28-35_SL8 variants linked to the barcode of lineage 176 were similar to those linked to barcodes of other lineages present during acute infection (e.g., 5836, 4221, and 630) (Fig 6B). However, these other barcoded virus lineages disappeared from the population, despite being linked to the same Tat_28-35_SL8 variants as 176 at three weeks post-infection (Fig 6B). These data cumulatively suggest that additional sequences outside of Tat_28-35_SL8 in lineage 176 contributed to its expansion during chronic infection, while the other lineages disappeared.

## Discussion

In this study, two cynomolgus macaques were challenged IV, and two rhesus macaques were challenged IR with 200,000 IUs of SIVmac239M. Assessing lineage-specific viral dynamics within the viral population increases the power to make conclusions despite the small number of animals. Here we found between 70–560 times more viral lineages circulating during acute infection in the IV-challenged macaques than in the IR-challenged macaques. This difference in the number of unique viral lineages is consistent with historical studies showing that infection route influences the number of virus lineages circulating systemically soon after infection with swarm virus populations [36]. The number of unique virus lineages present after IR challenge in our study was similar to those detected in animals challenged IV with 100 IUs of SIVmac239M [3].

Previous studies comparing the number of transmitted viruses between different challenge routes were complicated by sequence variability in the virus population [36]. One previous study compared challenge routes with SIVmac239X, a stock containing only ten different lineages [1], thereby drastically limiting the power to detect differences in high dose settings. Here, we compared the number of lineages circulating after IR vs. IV challenge with a challenge stock containing thousands of unique viral lineages, called SIVmac239M. Similar to challenging animals with a swarm virus, we found reduced transmission of virus lineages upon intrarectal challenge with SIVmac239M, which is consistent with the hypothesis that the challenge route is the primary driver determining the number of individual virus lineages that initiate systemic SIV/HIV replication [36,37].

Other studies of antiretroviral-naïve macaques challenged IV with SIVmac239M tracked virus populations only up to 32 days post-infection [8]. In that study, they found that the virus population had drastically changed with at least a 3-fold change in detectable barcodes at day 32 [8]. Building on that study, we tracked the virus population up to 84 days post-infection in the two intravenously challenged cynomolgus macaques. We found an even greater reduction in the number of systemic lineages in both cynomolgus macaques at approximately 21 days post infection. Further, after the detection of acute T cell responses, the number of unique viral lineages in the IV-challenged cynomolgus macaques decreased to below 50. The species, depth of sequencing, time points selected, or prior experience of the animals are all possible reasons for the increased reduction in the number of virus lineages circulating in cynomolgus macaques. Future studies comparing SIVmac239M dynamics in both of these species will be needed to identify which factors may be responsible for these differences.

In contrast to the animals challenged intravenously, there were too few virus lineages for the animals challenged intrarectally to accurately dissect out whether an early variant in a targeted epitope emerged on a single or on multiple barcoded virus lineages. However, we noticed that even the small number of barcode lineages detected in the rhesus macaques declined into chronic infection, suggesting that some lineages were being eliminated. Because we captured the molecular barcode and the Tat_28-35_SL8 T cell epitope sequence on the same amplicon, we tracked the persistence or elimination of particular combinations of barcodes and epitope sequences. We found that multiple independent virus lineages contained the same nucleotide mutations in the sequence coding for Tat_28-35_SL8 from three to six weeks post-infection, consistent with a previous study of rhesus macaques infected IV with SIVmac239M (8). When sequencing viruses into chronic infection, we found that one barcode lineage expanded to comprise the majority of the virus population and was linked to the same diverse array of mutations in Tat_28-35_SL8. In contrast, the other barcode lineages that had been present three weeks after infection and linked to the same Tat_28-35_SL8 variants reduced in frequency or disappeared from the population by necropsy (Figs 3 and 6).

We were surprised that not all of the barcode lineages survived into chronic infection in the rhesus macaques even when they shared the same variant sequences in Tat_28-35_SL8 during acute infection. By continuing to follow the virus lineages into chronic infection, we suggest that surviving viral lineages have an additional mechanism offering them a fitness advantage. One hypothesis is that mutations elsewhere in the viral genome conferred this advantage. However, mutations in Tat_28-35_SL8 are known occur very early after infection (8), so it is unlikely that another variant emerged on the same lineage before mutations arose in Tat_28-35_SL8. An alternative hypothesis is that there was local expansion of a single barcode lineage that allowed it to dominate. A previous study of vaginal infection with SIVmac239X suggested that there were different groups of viruses localized to different foci in the tissues soon after infection (13). One could imagine that local inflammation at a tissue site with a specific barcode lineage may induce lineage expansion that is unrelated to variant epitope sequences. Future studies that examine the sequence and localization of individual viral lineages in SIVmac239M-infected macaques may help reveal additional mechanisms of viral persistence.

Overall, this study revealed some exciting results that may expand future uses for SIVmac239M. We found that strong, early CTL responses eliminated the majority (97%) of viral lineages in CMs. This data sets the stage for future studies that could explore whether augmentation of acute CTL responses (e.g. vaccination or activation) might be able to reduce virus lineages. This unique model allows for unprecedented resolution of viral population dynamics. With this large dynamic range, it is possible that interventions could be optimized so that even small improvements are detectable and build upon current methods.

In addition, the difference in virus dynamics between the IV-challenged cynomolgus macaques studied here versus previous studies of Mamu-A1*001+ rhesus macaques infected intravenously with SIVmac239M suggests that perhaps the acute selection mechanisms of virus lineages may vary by species or host MHC genotype. It would be interesting to compare the selection of acute-phase barcode lineages between Mamu-A1*001+ and Mamu-B*08+ rhesus macaques challenged with SIVmac239M to determine how the quality of the earliest T cells affects the population of virus lineages present during acute infection. Further, additional studies comparing SIVmac239M replication dynamics between rhesus macaques and other macaque species may identify critical features of species-specific SIV pathogenicity that are not apparent when animals are challenged with clonal SIVmac239.

Lastly, even though our study only challenged two animals intrarectally with SIVmac239M, our data is consistent with other studies finding that virus transmission is restricted by mucosal tissue [36]. Future studies that continue to explore how the number of transmitted SIVmac239M lineages by different routes may better quantify how mucosal tissue limits transmission under different types of stressors.

## Conclusions

In this study, we conclude that virus lineages present during acute infection can still be eliminated from the detectable plasma viral population even after initial selection by acute T cell responses. Additionally, we suggest that the longitudinal success of one virus lineage over others is likely due to factors besides variation in T cell epitopes targeted during acute SIV infection. These conclusions provide new insights into the characteristics of viral lineage survival during acute and chronic SIV infection of macaques.

## Supporting information

S1 Fig Schematic of DNA amplicons generated for sequencing. (A) A schematic of the relative locations of the 4 RT-PCR amplicons generated to sequence the complete SIV genome. (B) A schematic of the PCR amplicons generated to sequence the 34 nt barcode region. The long amplicon was generated by RT-PCR with primers 5942F and 8184R. The subsequence two PCR amplicons are then shown below. For both (A) and (B), the location of the 34-nucleotide barcode is noted, as well as the three CD8 T cell epitopes of interest.
(TIF)

S1 Table RT-PCR and PCR primers used for study. RT-PCR primers and PCR primers used for r10001, r04103, cy0428, and cy0575 are listed. Replicates for cy0428 and cy0575 were separated following vRNA isolation from plasma, while replicates for r10001 and r04103 were separated following RT-PCR. Replicates were included wherever they were completed. PCR primers contained Common Sequence (CS) tags. Primer sequences, along with two different common sequence tags, were incorporated in different variations.
(XLSX)

## Abbreviations

HIV: Human Immunodeficiency Virus
SIV: Simian Immunodeficiency Virus
IV: Intravenous
IR: Intrarectal
IU: Infectious units
SGA: Single Genome Amplification
ART: Antiretroviral therapy
CM: Cynomolgus macaque
RM: Rhesus macaque
WNPRC: Wisconsin National Primate Research Center
VC: Viral copies
VL: Viral loads
Ceq: log viral copies
CS: Common sequence
dsDNA: double stranded DNA
HS: high sensitivity
PBMC: Peripheral blood mononuclear cells
ELISPOT: Enzyme linked immunosorbent spot
SFC: spot forming cell
SDI: Simpson’s Diversity Index
