# Supplementary figures and images for: The mucosal barrier and anti-viral immune responses can eliminate portions of the viral population during transmission and early viral growth
Source: PLoS One. 2021 Dec 2;16(12):e0260010. doi: 10.1371/journal.pone.0260010 (PMC8639003; doi:10.1371/journal.pone.0260010)

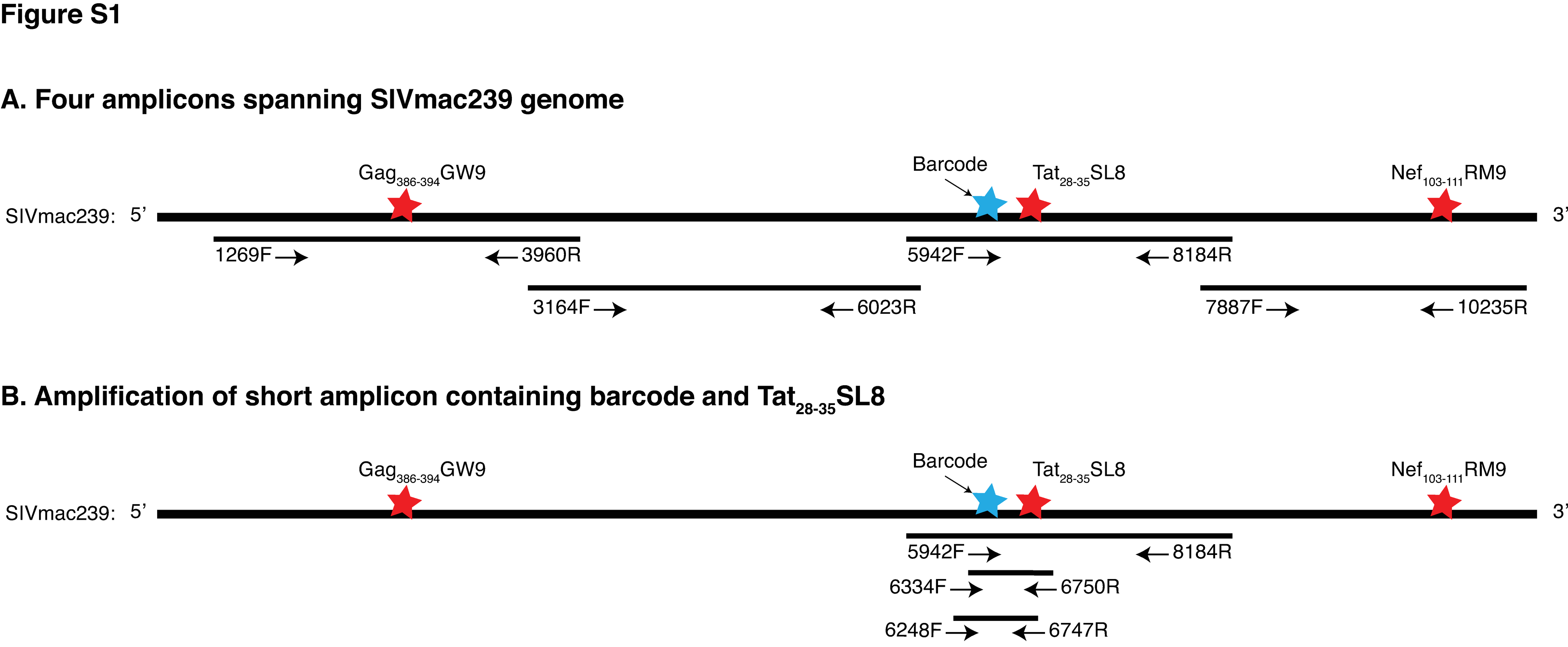

Supplement: S1 Fig — (A) A schematic of the relative locations of the 4 RT-PCR amplicons generated to sequence the complete SIV genome. (B) A schematic of the PCR amplicons generated to sequence the 34 nt barcode region. The long amplicon was generated by RT-PCR with primers 5942F and 8184R. The subsequence two PCR amplicons are then shown below. For both (A) and (B), the location of the 34-nucleotide barcode is noted, as well as the three CD8 T cell epitopes of interest. (TIF) [file pone.0260010.s001.tif]
